# Adherence to Tuberculosis Treatment among Migrant Pulmonary Tuberculosis Patients in Shandong, China: A Quantitative Survey Study

**DOI:** 10.1371/journal.pone.0052334

**Published:** 2012-12-17

**Authors:** Chengchao Zhou, Jie Chu, Jinan Liu, Ruoyan Gai Tobe, Hong Gen, Xingzhou Wang, Wengui Zheng, Lingzhong Xu

**Affiliations:** 1 Institute of Social Medicine and Health Service Management, School of Public Health, Shandong University, Jinan, China; 2 Shandong Centre for Disease Control and Prevention, Jinan, China; 3 HealthCore, Inc. Wilmington, Delaware, United States of America; 4 Shandong Center for Tuberculosis Prevention and Control, Jinan, China; Institut de Pharmacologie et de Biologie Structurale, France

## Abstract

Adherence to TB treatment is the most important requirement for efficient TB control. Migrant TB patients’ “migratory” nature affects the adherence negatively, which presents an important barrier for National TB Control Program in China. Therefore, TB control among migrants is of high importance.The aim of this study is to describe adherence to TB treatment among migrant TB patients and to identify factors associated with adherence. A total of 12 counties/districts of Shandong Province, China were selected as study sites. 314 confirmed smear positive TB patients were enrolled between August 2^nd^ 2008 and October 17^th^ 2008, 16% of whom were non-adherent to TB therapy. Risk factors for non-adherence were: the divorced or bereft of spouse, patients not receiving TB-related health education before chemotherapy, weak incentives for treatment adherence, and self supervision on treatment. Based on the risk factors identified, measures are recommended such as implementing health education for all migrant patients before chemotherapy and encouraging primary care workers to supervise patients.

## Introduction

China ranks second on the list of countries with the highest number of estimated tuberculosis (TB) patients [1]. Migrants, numbering more than 210 million nationwide [2], suffer a disproportionate burden of disease due to TB in China. As reported in many studies in China, migrants have greater contribution to keeping higher TB incidence than that of local residents [3]–[6]. Due to usually low education level, weak awareness of TB and poor financial conditions, treatment outcomes among migrant pulmonary tuberculosis (PTB) patients were much worse when compared to the local patients, which presents an important barrier for National TB Control Program in China [7]–[8]. Therefore, TB control among migrants is of high importance.

Adherence to TB treatment is the most important requirement for efficient TB control [9]. TB treatment presents particular challenges for adherence because a standard treatment lasts 6 or 8 months and involves taking a number of medications, and side-effects are common during the treatment [10]. Studies conducted in Jiangsu Province and Fujian Province of China indicated that the percentage of non-adherence among permanent residents was lower than that among migrants [11]–[12]. Compared to the permanent residents, migrant PTB patients’ “migratory” nature affects negatively the adherence [13]. Poor adherence to TB therapy is regarded as the most common cause of treatment failure and disease relapse, which contributes to patient morbidity, mortality, transmission and drug resistance [14]–[15].

Efforts to improve treatment outcomes require a better understanding of the barriers to and facilitators of adherence to TB treatment. Studies conducted in China show that a number of factors associated with non-adherence include the health system, patient-related and socio-economic factors [11], [16]–[17]. Most of the studies have been done only among permanent patients. Studies on adherence among migrant PTB patients, however, are limited in number. It is therefore important to identify determinants of adherence to TB treatment among migrants.

**Table 1:** Univariate analysis of the risk factors for non-adherence to TB treatment among migrant PTB patients.

Variables	non-adherence		adherence		OR	95%CI	*P*
	n	%	n	%			
**Sex**							0.291
Male	35	17.6	164	82.4	1.0	–	
Female	15	13.0	100	87.0	0.70	0.37–1.35	
**Age(in years)**							0.092
15∼29	26	15.4	143	84.6	1.0	–	
30∼44	21	21.2	78	78.8	1.48	0.78–2.80	
≥45	3	6.5	43	93.5	0.38	0.11–1.33	
**Educational level**							0.074
Primary school and below	14	23.7	45	76.3	1.0	–	
Junior school	22	17.5	104	82.5	0.680	0.32–1.45	
Senior school and above	14	10.9	115	89.1	0.391	0.07–1.19	
**Marital Status**							**0.017**
Single	14	10.0	126	90.0	1.0		
Married	32	19.6	131	80.4	2.198	1.12–4.31	
Divorced/bereft of spouse	4	36.4	7	63.6	5.143	1.34–19.78	
**Household origin**							0.178
Rural	47	17.0	230	83.0	1.0		
Urban	3	8.1	34	91.9	0.43	0.13–1.47	
**Household income level**							0.904
Lowest group	11	13.9	68	86.1	1.0		
Lower group	16	15.7	86	84.3	1.15	0.50–2.64	
Higher group	12	16.2	62	83.8	1.20	0.49–2.91	
Highest group	11	18.6	48	81.4	1.42	0.57–3.53	
**Debt status**							**0.001**
No	25	11.2	199	88.8	1.0		
≤10000 Yuan(RMB)	14	24.6	43	75.4	2.59	1.25–5.39	
>10000Yuan(RMB)	11	33.3	22	66.7	3.98	1.73–9.17	
**Working hours per day**							**0.025**
≤8	26	12.6	181	87.4	1.0		
>8	24	22.4	83	77.6	2.01	1.09–3.71	
**Working days per week**							**0.049**
≤5	15	11.2	119	88.8	1.0		
>5	35	19.4	145	80.6	1.92	1.01–3.67	
**Health insurance status**							0.389
Yes	15	13.5	96	86.5	1.0		
No	35	17.2	168	82.8	1.33	0.69–2.57	
**Distance to local CTD (Kms)**							0.783
0-	10	13.3	65	86.7	1.0		
5-	10	14.1	61	85.9	1.07	0.42–2.74	
10-	20	18.3	89	81.7	1.46	0.64–3.33	
20-	10	16.9	49	83.1	1.33	0.51–3.44	
**Knowledge about TB before diagnosis**							**0.002**
Yes	8	7.0	106	93.0	1.0		
No	42	21.0	158	79.0	3.52	1.59–7.80	
**TB-related health education before chemotherapy**							**0.001**
Yes	32	12.5	224	87.5	1.0		
No	18	31.0	40	69.0	3.15	1.62–6.15	
**Patients evaluation on free treatment policies incentiveness to adherence**							**0.000**
Strong	29	11.3	227	88.7	1.0		
Moderate	5	22.7	17	77.3	3.46	1.32–9.10	
Weak	16	44.4	20	55.6	6.73	3.12–14.51	
**Treatment supervisor**							**0.001**
Self supervision	25	28.4	63	71.6	1.0		
Family members	16	16.8	79	83.2	0.51	0.25–1.04	
Primary care physicians	4	4.8	80	95.2	0.13	0.04–0.38	
Volunteers	5	10.6	42	89.4	0.30	0.11–0.85	

* **:Household income level:** 1) Lowest group (≤10000 RMB Yuan per year); 2) Lower group(10001–20000 RMB Yuan per year); 3) Higher group (20001–30000 RMB Yuan per year ); 4) Highest group (>30000 RMB Yuan per year).

The objective of this study is to describe adherence to TB treatment among migrant PTB patients in Shandong, China, and to identify factors associated with adherence, so as to provide an evidence for the development and improvement of TB control strategy among migrants in China.

## Methods

### Study Site

This study was conducted in Shandong Province, the second largest province in China. Shandong consists of 17 municipalities and 140 counties (districts) with a total population of nearly 100 million. By the end of 2008, Shandong had about 6.91 million migrants. Direct observed therapy, short course (DOTS) strategy for TB was introduced in the 1990s and is now 100% available in all counties (districts) in Shandong province. County TB dispensary (CTD) is the authorized institution providing TB diagnosis, treatment and cases management, and all newly diagnosed TB cases are required to be registered in the local CTD and reported to upper level health authorities.

**Figure 1:**
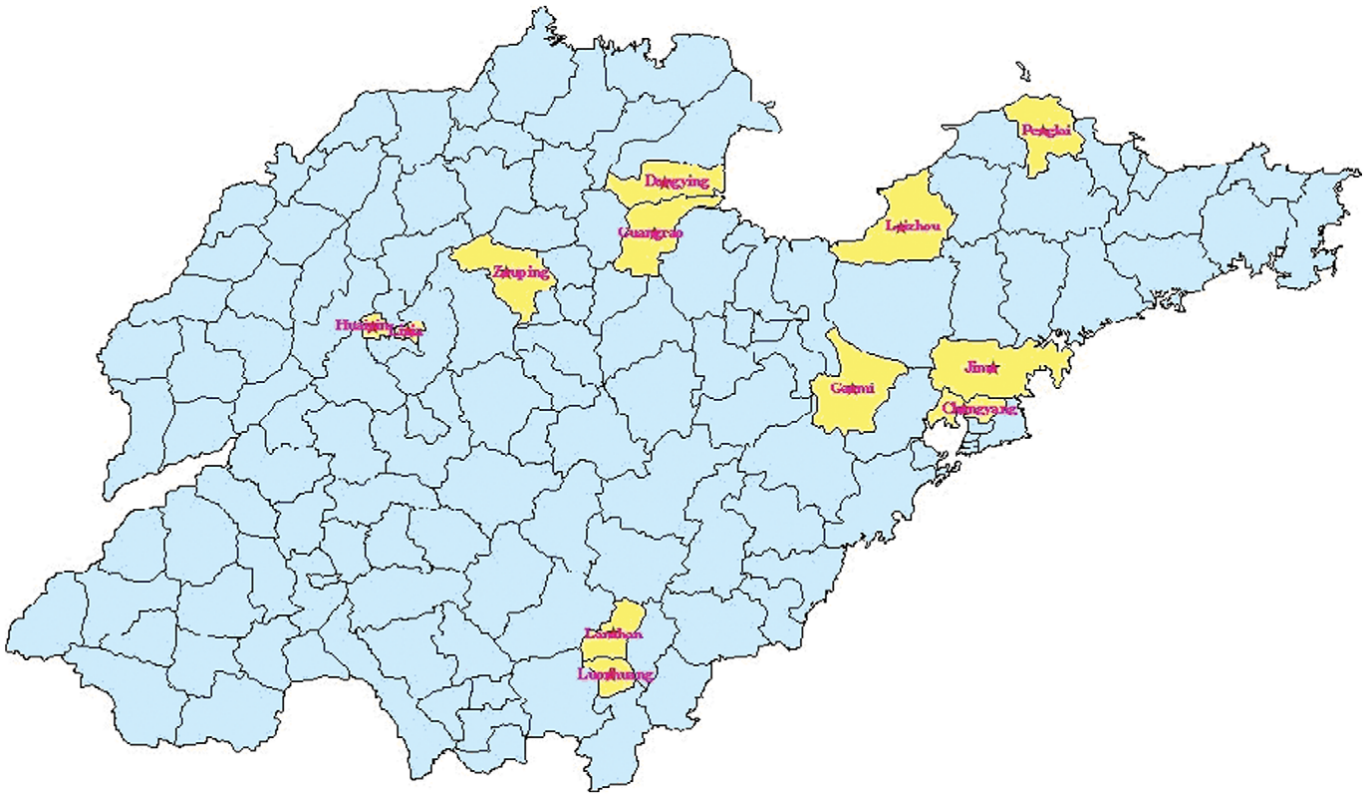
Location of the 12 study sites in Shandong province.

A total of 12 counties/districts (including Huaiyin District, Lixia District, Dongying District, Guangrao County, Penglai County, Laizhou County, Zouping County, Chengyang District, Jimo County, Luozhuang District, Lanshan District, Gaomi County) were selected as study sites after considering the larger number of migrants in these counties (districts) and the feasibility of the study implementation. The Figure 1 shows the location of the 12 study sites in Shandong province.

**Table 2:** Final model of the risk factors for non-adherence to TB treatment among migrant PTB patients.

Variables	OR _adj_	95%CI	*P*-value
**Marital Status**			
Single	1.0		
Married	2.64	1.15–6.05	**0.022**
Divorced/bereft of spouse	2.87	0.52–15.81	0.226
**Debt status**			
No	1.0		
≤10000 Yuan(RMB)	1.86	0.74–4.47	0.185
>10000Yuan(RMB)	1.95	0.67–5.72	0.224
**Working hours per day**			
≤8	1.0		
>8	1.35	0.63–2.91	0.438
**Working days per week**			
≤5	1.0		
>5	1.38	0.60–3.17	0.453
**Knowledge about TB before diagnosis**			
Yes	1.0		
No	1.98	0.78–4.93	0.154
**TB-related health education before chemotherapy**			
Yes	1.0		
No	2.53	1.01–6.36	**0.049**
**Patients evaluation on free treatment policies incentiveness to adherence**			
Strong	1.0		
Moderate	1.87	0.47–7.46	0.375
Weak	6.03	1.89–19.29	**0.002**
**Treatment supervisor**			
Self supervision	1.0		
Family members	0.39	0.14–1.02	0.055
Primary care physicians	0.15	0.04–0.51	**0.003**
Volunteers	0.36	0.11–1.16	0.087

### Study Participants

The participants must meet the following selection criteria: 1) They were smear-positive pulmonary migrant TB patients registered in CTD of the sampling sites during the period from August 1^st^ 2007 to July 31^st^ 2008; 2) If the patients were being treated, the treatment time must be over 1 month; 3) If the patients had finished normal treatment, the closure must happen within 6 months. There were 314 patients finally recruited in this study.

### Data Collection

A cross-sectional study was conducted between August 2^nd^ 2008 and October 17^th^ 2008. All the participants were interviewed face-to-face at local CTD using self-making survey tool, which included demographic characteristics information of the participants, TB-related health seeking behavior and adherence to TB therapy. The interviews were undertaken by trained postgraduates from School of public health of Shandong University and physicians from Shandong Centre for TB Prevention and Control.

### Definition of Migrants, Adherence

In this study, “migrants” were non-local residents who had lived or planned to live in a certain area for more than 3 months [18]. Adherence refers both to taking drugs according to prescription and to attending follow-up check-ups. We consider it as non-adherence if the patients have at least 1 missed medication or follow-up medical appointments during the TB treatment.

### Data Analysis

The data was double entered using EPI Data 6.04, and analyzed using SPSS 13.0 for Windows. Descriptive and inferential analyses were performed as appropriate. Univariate logistic regression was used to analyze the association of each variable with adherence to TB treatment. Multi-logistic regression model was developed to further assess the impact of variables on adherence. The magnitude of the association was measured by the odds ratio (OR) with confidence interval (CI). A P-value of <0.05 was considered statistically significant.

### Ethical Consideration

The Ethical Committee of School of Public Health of Shandong University approved this study. All participants aged 18 and above provided written informed consents to participate in the study. As for participants under 18 years, we obtained written informed consents from the guardians on behalf of them (only 3 of the 314 participants were under 18 years). The investigation was conducted after the informed consents of all participants were obtained.

## Results

A total of 314 confirmed smear positive PTB patients were enrolled in a 2.5 months study period. The age of the study participants ranged from 15t o 70 years, with a mean age of 31.8 years. About 63% were the male. With regards to education, 19% were with primary education or illiterate, 40% with junior education and 41% with senior education and above. 45% of the respondents were single (never-married), while 52% were married, 4% divorced or widowed. 65% of the patients did not have any kind of health insurance.

We observed that 16% of the subjects experienced non-adherence during our study.The adherence was compared among different subgroups using univariate logistic regression analysis. It was found that those patients who were divorced or bereft of spouse (***P***<0.05), who had a debt over RMB 10,000 Yuan (***P***<0.01), who worked over 8 hours per day (***P***<0.05) and 5 days per week(***P***<0.05), who had no knowledge about TB before diagnosis (***P***<0.05), who had not received TB-related health education before chemotherapy (***P***<0.01), whose evaluation on free policies incentives to adherence was weak(***P***<0.01),who supervised themselves on treatment (***P***<0.01 ) were more likely not to adhere to TB treatment (Table 1).

Multivariate logistic regression model was employed to analyze risk factors associated with non-adherence, and also to control possible confounding of the influences of the factors. The output showed that patients’ marital status, whether received TB-related health education before chemotherapy, patients evaluation on free treatment policies incentives to adherence and treatment supervisor were factors significantly associated with non-adherence. None of the others entered the model (Table 2).

## Discussion

Providing effective treatment is a key intervention to prevent the spread of TB among migrants. However, a long-term TB treatment could easily lead to a non-adherence [19]–[20]. In our study, 16% of the participants did not adhere to the TB treatment. The percentage of non-adherence found in our study was higher than the 10% which was required to achieve treatment success of 85%, one of the health-related indicators of the Millennium Development Goals (MDGs) [21]. It was also higher than the 8.9% reported in the patients among permanent residents in the same province [22], which is similar to the findings conducted in Jiangsu Province of China [11]. The high percentage of non-adherence among migrants may reflect the fact that there are more quality problems in DOTS implementation among migrants than permanent residents.

We found that being divorced or bereft of spouse was a risk factor for non-adherence to TB treatment among migrants. The divorced or bereft of spouse would lack of support from the spouse, therefore they shouldered more economic and social burdens after suffering from TB. Some studies indicated that family members played an important role in the treatment supervision [23]–[24]. As a core member of the family, the spouse would also observe patients taking medications, encourage and remind them to keep the follow-up medical appointments, so as to complete the TB treatment successfully. There was one point to note was that the sample size of divorced/bereft of spouse(only 11 persons) was very small, which would make the output a significant change.

Non-adherence to TB treatment often results from inadequate knowledge of the treatment regimen and importance of adherence [25]–[26]. Therefore, *National TB Control Program Implementation Guide in China (2008 Edition)* put forward clearly that carrying out health education for the TB patients before chemotherapy was the most important step of DOTS implementation, and the treatment regimen and importance of adherence were the core knowledge of the education. In our study, patients who had received TB-related health education before chemotherapy were more likely to be compliant. Similar findings were observed in other countries [24], [27]–[28]. Consequently, the local CTD should implement health education for all migrant patients before chemotherapy.

Chinese government had implemented policy to provide TB drugs, free chest X-ray test for PTB patients, and it also covered migrant patients. However, the free policy did not necessarily motivate patients to adhere to the treatment. One possible reason was that there were also some items being not covered in the free package, such as follow-up check-ups and transport costs occurring in the care-seeking process, which probably weaken patients’ satisfaction, and consequently affect negatively their adherence. Therefore the government should expand the free package for migrant TB patients.

Similar to the findings of other studies in China, Nepal and India [12], [29]–[30], primary health care workers’ supervision was a protective factor to migrant patients’ adherence, and primary care physicians’ frequent attendance in the treatment supervision would facilitate patients’ adherence. This finding should therefore give an impetus to encourage primary care workers to supervise patients, especially during the intensive phase of DOTS.

One limitation of this study was that the selection of migrant PTB patients might be biased. Patients who were not detected or had no registration in local CTD or had left the sample sites during the period of the survey were not included. Another limitation was that the measure of non-adherence was self-reported, so recall bias, bound to impose a certain effect on the study, was unavoidable. Furthermore, the small sample size of divorced/bereft of spouse might have an impact on the significance of the result. Despite these limitations, the current study has several strengths. First, the questionnaire was developed using a range of scientific methodologies including literature reviews, focus groups, and pilot testing. Second, the study included relatively large numbers of migrant PTB, which enabled a more close examination of the influencing factors of adherence issue.

### Conclusion

This study has made attempts to provide insight into adherence to TB treatment and its determinants among migrant TB patients in Shandong, China. The output showed that 16% of the subjects experienced non-adherence. Factors affecting non-adherence included patients’ marital status, whether received TB-related health education before chemotherapy, patients evaluation on free treatment policies incentives to adherence and treatment supervisor. More importance should be given to non-adherence to TB treatment among migrant TB patients. Interventions such as implementing health education for all migrant patients before chemotherapy and encouraging primary care workers to supervise patients would be urgently required to lead to good adherence to TB treatment among migrants.
